# Climate Change and Severe Drought Impact on Aflatoxins and Fungi in Brazil Nuts: A Molecular Approach

**DOI:** 10.3390/ijms26199592

**Published:** 2025-10-01

**Authors:** Ariane Mendonça Kluczkovski, Janaína Santos Barroncas, Hanna Lemos, Heloisa Lira Barros, Leiliane Sodré, Liliana de Oliveira Rocha, Taynara Souza Soto, Maria Luana Vinhote, Augusto Kluczkovski

**Affiliations:** 1 Faculty of Pharmaceutical Sciences, Federal University of Amazonas, Manaus 69067-005, Brazil; janaina.barroncas@gmail.com (J.S.B.); hannabarbosale@gmail.com (H.L.); heloisalbarros20@gmail.com (H.L.B.)mluanaaraujo@hotmail.com (M.L.V.); 2 Faculty of Food Engineering, University of Campinas, Campinas 13083-970, Brazil; lrocha@unicamp.br (L.d.O.R.); t203097@dac.unicamp.br (T.S.S.); 3 Foundation in Health Surveillance-Amazonas, Products Management Section, Manaus 69093-018, Brazil; augustokjr@hotmail.com

**Keywords:** aflatoxin, *Aspergillus* sp., *Bertholletia excelsa*, mycotoxins

## Abstract

The Brazil nut production chain, which is reliant on Amazonian environmental conditions, is significantly affected by climate change, particularly extreme droughts, which decrease production and compromise sanitary quality. This study evaluated the influence of severe drought on aflatoxin concentrations and sequence toxigenic fungi in Brazil nuts harvested during the 2023 off-season. Aflatoxins were quantified using high-performance liquid chromatography, while fungal sequencing involved DNA extraction, PCR, and sequencing analysis. Findings indicated that all Brazil nut samples collected during extreme drought contained detectable aflatoxins, with 10% exceeding the legal threshold of 10 µg/kg. Phylogenetic analysis identified four isolates as *Penicillium citrinum*. Additional morphological and sequencing analyses identified *Aspergillus* species from the *Circumdati* and *Flavi* sections, although one isolate could not be taxonomically classified. These results demonstrate the aflatoxin production by fungi in Brazil nuts in an unprecedented way under drought conditions. Furthermore, the diversity of fungal species during drought underscores the risk of contamination, emphasizing the necessity for monitoring future harvests to improve management and ensure product safety.

## 1. Introduction

Intense climate events have impacted global food production and sanitary quality. Environmental conditions can modify the metabolism of microorganisms and influence food contamination by toxic agents, such as mycotoxins. Consequently, mitigation strategies are essential to protect consumer health. Anthropogenic activities represent a major driver of climate change, which are gradually destabilizing the equilibrium between plant growth and fungal diseases [1]. Factors such as temperature, water availability, and light quality and quantity can influence droughts and floods, impacting food production, fungal metabolism, and their toxin production capabilities [2]. Research on mycotoxigenic fungal responses to climate conditions has *Aspergillus*, *Penicillium,* and *Fusarium* species as primary global concerns due to their production of aflatoxins (AFL), ochratoxin A, and fumonisins. Mycotoxins are toxic agents to humans and cause hepatotoxicity, teratogenic effects, and cancer. Fungal contamination by these toxic agents disrupts the global food economy and trade [3,4,5].

In Brazil, efforts to achieve sustainable food production face the challenge of adapting to climate change to maintain sanitary standards and minimize economic losses. For instance, the Brazil nut (*Bertholletia excelsa*), a tropical oilseed harvested from extractive activities in tropical forests across several countries, is directly dependent on environmental conditions. The tree absorbs nutrients from the soil through its roots and can reach heights of up to 50 m [6]. Brazil nuts are rich in protein, fat, selenium, and other minerals, providing nutritional benefits for humans and animals. Their fruits are collected from the ground during the rainy season (December to April), depending on the region, and then ground, stored, and transported for processing. The predominantly artisanal nature of Brazil nut harvesting lacks technological systems, resulting in sanitary vulnerability to environmental and climate changes. These conditions can promote fungal proliferation and subsequent AFL production during various production stages.

Fungi that produce toxins can also be influenced by variables intrinsic and extrinsic to the food climate, which significantly influence toxin production and have been extensively studied in foods across multiple countries [7,8,9]. In the Brazil nut supply chain, temperature directly influences AFL production by certain *Aspergillus* spp. Combining molecular and physiological data facilitates understanding of aflatoxigenic species’ responses to temperature changes, enabling the prediction of environmental factors that affect AFL contamination in Brazil nuts [10]. Brazil nut trees generally grow in environments with annual mean temperatures of 23.5–27.6 °C and annual rainfall ranging from 1445–3399 mm [11]. These environmental conditions favor AFL production by some toxigenic fungi. For example, AFL can be detected in fresh, unprocessed, in-shell Brazil nuts stored at 27 °C and relative humidity (RH%) of 97.90 and 80% [12].

In Africa, extreme drought has increased the presence of mycotoxins in food. Other studies have found a significant correlation between increased AFL concentrations and insect-damaged crops. Warmer years would automatically lead to an increased risk of contamination. Contributing factors such as mycobiota profile and interactions, differences in each crop, and interaction with a constantly changing climate seem to contribute to contamination. Drought conditions have been related to an increase in aflatoxin in several crops when the fungi’s secondary metabolism increases the toxin production [13,14]. Therefore, predictive studies employing climate models with variables including precipitation, RH%, and temperature are required to assess the effects that climate change may have [15,16]. In Malawian maize, the changes are projected to make pre-harvest conditions more favorable for AFL contamination, and risk maps have been developed for these scenarios [17]. In Brazil, analyses of climate change impacts on mycotoxin presence in coffee revealed direct effects on the coffee microbiota, as crops are sensitive to water scarcity and heat stress. Higher temperatures have reduced product quality, with AFL, which are more toxic than ochratoxin A (OTA), promoting greater food insecurity in coffee production [18]. Brazil nuts are particularly vulnerable to AFL contamination due to their increased soil contact, as they are harvested through extractive activities in the Amazon rainforest. These nuts rely on rainfall for natural growth and are not cultivated, which increases dependency on environmental conditions. For example, in 2019–2020, a previous work showed undetected levels of AFL in dried samples and low levels of AFL in undried samples from both harvest years. Samples met the acceptable limit for shelled Brazil nuts [19]. Extreme drought conditions associated with climate change since 2023 may influence AFL concentration. In Brazil, nut production has declined since 2023, and further decreases are anticipated even though official data from the Brazilian Institute of Geography and Statistics has not yet been released. This reduction is likely to negatively impact not only commercial aspects but also health quality. Thus, this study evaluated the occurrence of contamination by fungi and AFL and sequenced toxigenic strains in Brazil nut samples obtained during the 2023 extreme drought.

## 2. Results

A screening of Brazil nut samples collected during the 2023 extreme drought of the 2023 harvest was conducted to assess the incidence of AFL contamination and to identify fungal species that may produce AFL.

### 2.1. Aflatoxins, Water Activity, and Moisture Content

During the 2023 extreme drought, approximately 10% (*n* = 2) of the samples exhibited total AFL concentrations exceeding the legal limit of 10 µg/kg, and all samples contained at least one type of AFL (B1, B2, G1, or G2). The measured AFL concentrations are presented in Table 1.

The mc% results are within maximum levels (<15%) as per the Brazilian legal limit (Table 2) [21]. However, approximately 90% of the samples exhibited Aw levels > 0.7, as recommended by CAC [20]. The Aw results could be explained by the fact that some of the communities do not apply good management procedures (GMP), such as transport and storage with physical barriers against rain, and rapid collection of the pods to send for processing. These could be some of the preventive measures to prevent Aw > 0.70.

### 2.2. Molecular Analysis

**Figure 1:**
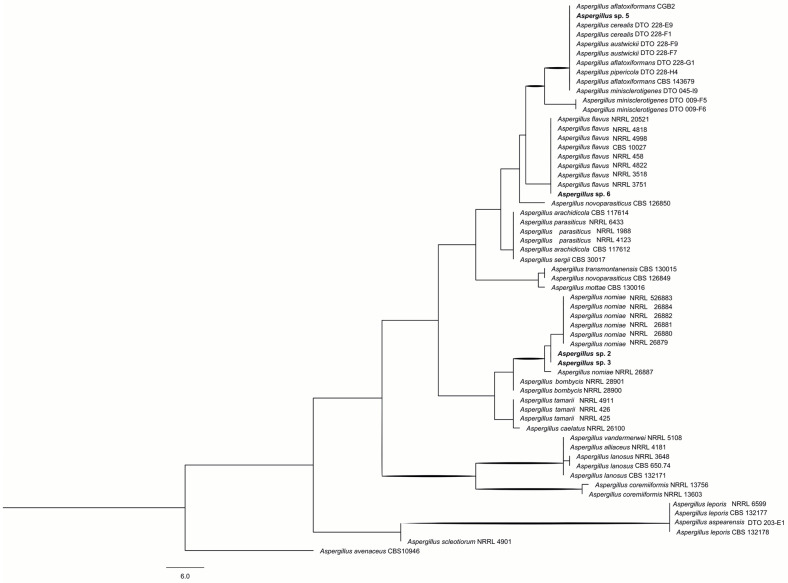
Phylogeny of *A.* section *Flavi* isolates based on combined ITS and *BenA* loci. Bootstrap values > 70% are indicated in bold. *A. muricatus* was used as the outgroup.

Isolates identified as *Aspergillus* through morphological analysis underwent further phylogenetic analysis using combined ITS and β-tubulin datasets. This analysis revealed that the strains belonged to two distinct sections, *Circumdati* and *Flavi* [22]. Within the *Flavi* section, isolates 2 and 3 formed a clade closely related to *A. pseudonomiae* and *A. nomiae*, while isolates 5 and 6 clustered within the *A. flavus* clade (Figure 1). The dataset for the *Flavi* section comprised 44 taxa and 844 characters, including 181 parsimony-informative characters (PICs). Phylogenetic analysis yielded nine most parsimonious trees (CI = 0.77, RI = 0.86).

**Figure 2:**
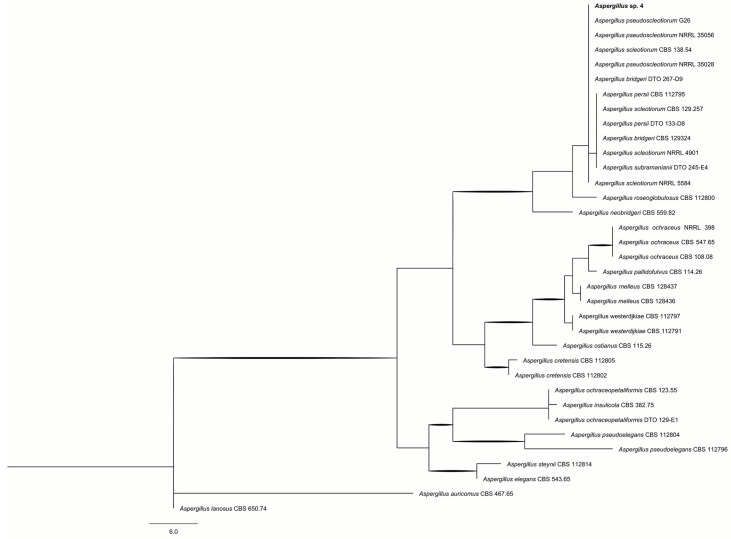
Phylogeny of *Aspergillus* section *Circumdati* isolates based on combined ITS and *BenA* loci. Branches with bootstrap values > 70% are indicated in bold. *A. tanneri* was used as the outgroup.

Members of the *Flavi* section are potential producers of mycotoxins, particularly AFLs. Within the *Flavi* series, *A. flavus* primarily produces AFL B1 and B2. In contrast, other species, such as *A. cerealis*, *A. austwickii*, *A. pipericola*, *A. minisclerotigenes*, and *A. aflatoxiformans*, are also capable of synthesizing AFL G and cyclopiazonic acid. In the *Nomiarum* series, another important group found in Brazil nuts [10], *A. nomiae* and *A. pseudonomiae* can produce both AFL B and G, as well as aspergillic acid, tenuazonic acid, and other extrolites [23,24]. The AFL are the most extensively studied mycotoxins regarding carcinogenicity in vertebrates. The AFL B and G present substantial health risks to humans and animals and commonly occur in fruits, cereals, and derived products. Additionally, derived forms of AFL B, M1, and M2 result from various metabolic processes and are present in milk and dairy products from individuals exposed to diets rich in AFL B. The harmful biological effects of AFL are diverse, including hepatotoxic, carcinogenic, mutagenic, and teratogenic effects. Isolate 4, belonging to the *Circumdati* section, clustered with *A. pseudosclerotiorum* in the *Sclerotiorum* series (Figure 2). The phylogenetic analysis comprised 43 taxa, with 244 PICs, yielding a single parsimonious tree (CI = 0.72, RI = 0.91). Species within this group produce aspernidines, cyclopenins, lovastatins, and sclerotiumins as major extrolites [25].

**Figure 3:**
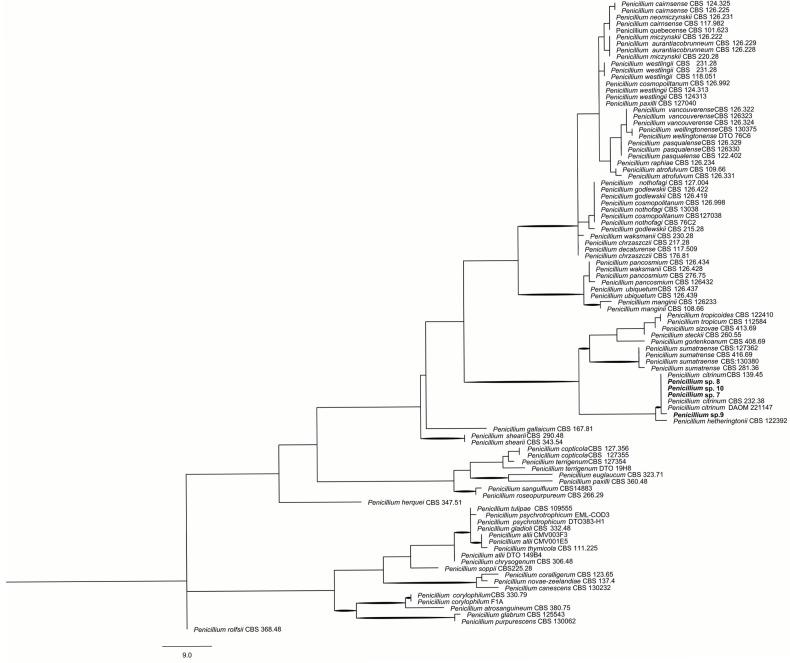
Phylogeny of *Penicillium* section *Citrina* isolates based on the ITS locus. Bootstrap values > 70% are indicated in bold. *P. rolfsii* was used as the outgroup.

Phylogenetic analysis of the ITS locus for isolates identified as belonging to the *Penicillium* genus through morphological examination indicated that isolates 7–10 (Figure 3) belong to the *P. citrinum*. The ITS dataset comprised 83 taxa and 488 characters, 87 of which were PICs. The phylogenetic analysis resulted in one parsimonious tree (CI = 0.60, RI = 0.89). Although the identification relied solely on the ITS locus, the study encompassed closely related species within the *Citrina* section. It demonstrated that the isolates clustered within the *P. citrinum* clade with high bootstrap support (>70%). *P. citrinum* is commonly found in temperate regions and occurs frequently in soil, food, and as an endophyte in coffee roots, stems, and leaves. Members of the *Citrina* section can produce the mycotoxins citrinin and citreoviridin, which are associated with nephrotoxic and neurotoxic effects, respectively [26].

## 3. Discussion

Research has reported that contamination of tree nuts by toxigenic fungi is primarily influenced by climatic, environmental, and processing factors [27]. Most existing studies on Brazil nuts have focused on agronomic properties and seed production, resulting in limited data available on quality and safety. Tree nuts are susceptible to AFL contamination under Aw ranges from 0.65 to 0.90, and seed MC% ranges from 14% to 22% [28]. The recommended threshold to prevent the toxigenic fungi and AFL development in Brazil nuts is Aw < 0.70 [20].

Previous research on AFL occurrence in raw Brazil nuts has reported lower AFL contamination compared to the observed results. Other studies found that 100% positivity was never recorded, regardless of the sample’s origin, whether from the field or the market [29,30]. For example, in the western and eastern Amazon regions, 80 samples collected directly from various forest sites during the 2006 harvest (a period without severe drought) were analyzed using liquid chromatography-tandem mass spectrometry. Total AFL concentration ranged from 1.2 to 11.5 µg/kg. Although the MC% and Aw from the samples did not show significant regional differences, the MC% was >20.0%, exceeding the legal limit of 15%. Similarly, the Aw level was 0.85 in both regions [31]. In another [19], only 2% of 23 samples from the 2019–2020 harvests tested positive for AFL, which were below the European Union regulatory limits [32]. The study assessed the effectiveness of drying steps through time/temperature parameters of MC% and Aw, and differences were observed in relation to Aw between the harvests and in the processed and unprocessed samples. Notably, processed samples exhibited a significant reduction in MC% compared to unprocessed ones, demonstrating the critical influence of adequate drying in preventing contamination.

Correlation was observed between severe drought events and increased AFL concentrations in the evaluated seeds. Stressful environmental conditions for microorganisms, such as low RH% and high temperatures (>30 °C), characteristics in the Amazon region during the 2023 harvest, lead fungi to perform secondary metabolism to conserve energy. This phenomenon prioritizes toxin production as an adaptive response, as fungal cells release these toxins upon cell death. Conversely, high rainfall levels increase the likelihood of *Aspergillus* growth, raising the risk of seed contamination.

Soil humidity promotes fungal growth, while drought induces metabolic stress in *Aspergillus*, stimulating toxin biosynthesis through secondary metabolism. The increased fungal proliferation and AFL contamination observed in the samples likely result from these interacting factors. Previous research has raised concerns and developed predictive models regarding the increase in AFL concentrations in tree nuts and other foods (e.g., maize) in response to climate change [33]. Therefore, the higher AFL concentrations detected during periods of lower rainfall in the study region may be directly related to climate change.

Concerning the *Penicillium* section, *Citrina* consists of species found in soil, marine samples, the human respiratory tract, and as contaminants in stored food. These species produce low levels of mycotoxins, particularly OTA and penicillic acid, which exhibit cytotoxic, immunotoxic, nephrotoxic, and potentially carcinogenic properties [34,35]. The occurrence of toxigenic strains was reported in association with climate change, which may directly and indirectly influence AFL proliferation. Increases in temperature and humidity may directly facilitate AFL production by providing favorable conditions for fungal growth. These variables are used to study their effects on sclerotia and sporulation in fungi, incorporating the resulting data into equations included in the AFL predictive model [35].

## 4. Materials and Methods

### 4.1. Sampling

Samples of raw Brazil nuts (*n* = 20), each weighing ≥ 3 kg, were obtained from seeds directly removed from pods. Samples were collected in October 2023 (off-season) from Amazonas State (northern Brazil), specifically Beruri (*n* = 10) and Itacoatiara (*n* = 10). Few communities had Brazil nuts available for sale at this period, as some regions experienced out-of-season pod falls. The raw Brazil nut samples were purchased directly from local or extractive communities and had not undergone industrial drying. Seeds were removed from pods and transported to the laboratory for analysis within 48 h. Samples exceeding 10.0 µg/kg (B1 + B2 + G1 + G2), which is the EU legal limit [20], were subjected to AFL for molecular analysis. Climate data were collected with calibrated data loggers (Instrutherm HT900^®^, São Paulo, Brazil) at the sampling stage.

In 2023, Amazonia experienced both historically dry and warm conditions. According to the Brazilian surveillance in climate [36], in October, temperature anomalies exceeded previous record values registered in 2015, being +3 °C above normal relative to the 1981–2020 mean. These historical dry and warm conditions are attributed to two key atmospheric mechanisms. The Amazon Basin reached record temperature values during 2023. From August to October, air temperature anomalies reached +1.8, +2.2, and +2.7 °C, respectively, since 1980, surpassing previous records [37]. During sample collection, the average temperature was 32 °C and the RH was 70%. Brazil nuts were not readily available, as it was off-season, and fresh Brazil nuts were rare outside of the regular harvest period. Therefore, the sample size was limited by reduced product availability.

### 4.2. Moisture Content and Water Activity

Moisture content (MC) was determined using an electronic moisture balance equipped with an infrared dryer (MOC-120H, Shimadzu, Barueri, Brazil) by drying approximately 1 g of the sample at 105 °C until all moisture evaporated [38]. Water activity (Aw) was determined at room temperature (25 °C) with a benchtop Aw instrument (Aqualab 4TE, DECAGON, São Paulo, Brazil) employing the dew point method. All analyses were conducted in triplicate, and results are presented as the mean ± standard deviation.

### 4.3. Aflatoxin Quantification

**Figure 4:**
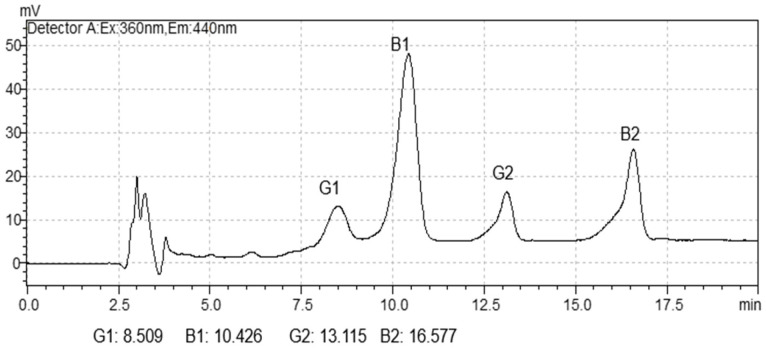
Chromatogram with the aflatoxin standards (B1, G1, G2 and B2).

AFL concentrations were quantified using high-performance liquid chromatography in accordance with the AOAC Official Method [39]. Samples (25 g) were extracted using 100 mL of acetonitrile:water (84:16 *v*/*v*), shaken at high speed for 3 min, followed by filtration through filter paper. Subsequently, 6 mL of the filtrate was transferred to a cleanup column (MULTISEP 226, Romer Labs, São Paulo, Brazil). From the purified extractions, 0.2 mL was removed and derivatized by adding 0.7 mL of a derivatizing solution (trifluoroacetic acid, glacial acetic acid, and distilled water) in a water bath at 65 °C. The derivatized sample was subjected to quantification in high-performance liquid chromatography with isocratic elution utilizing an acetonitrile, methanol, and ultrapure water (1:1:4) mobile phase on a Waters X-Terra column (4.6 mm × 150 mm), at a flow rate of 1.0 mL/min. A fluorescence detector with excitation at 360 nm and emission at 440 nm was used, with an injection volume of 50 μL and a total run time of 15 min. Three sets of AFL standards (B1, B2, G1, and G2; Sigma Aldrich) were employed. The limit of detection (LOD) and limit of quantification (LOQ) for AFB1, AFB2, AFG1, and AFG2 were 0.136, 0.136, 0.250, and 0.250 μg/kg (LOD) and 0.410, 0.410, 0.750, and 0.750 μg/kg (LOQ), respectively. The LOD was defined as a 3:1 signal-to-noise ratio, and the LOQ as a 10:1 ratio. The analytical curve was constructed with five points, each representing the average of five injections per extract, to determine the correlation coefficient (R) values for LOD and LOQ. Recovery rates for AFB1, AFB2, AFG1, and AFG2 were 93.5, 80.2, 98.5, and 98.1%, respectively. The AFL standards chromatogram is presented in Figure 4.

### 4.4. Molecular Analysis

#### 4.4.1. DNA Extraction, Polymerase Chain Reaction, and Sequencing Analysis of Housekeeping Gene ITS

Fungal cultures from samples positive for AFL toxin were evaluated from a total of 12 isolates. *Aspergillus* and *Penicillium* fungal cultures were grown on malt extract agar for 5 days at 25 °C. Genomic DNA was extracted using the Easy-DNA gDNA Purification Kit (Invitrogen, Thermo Fisher Scientific, Waltham, MA, USA) following the manufacturer’s instructions. The primer sets are illustrated in Table 3. The PCR was performed according to the method described by White et al. [40]. The resulting amplicons were purified with Exosap-IT (Thermo Fisher Scientific, Waltham, MA, USA) and submitted to the Central Laboratory for High-Performance Technologies in Life Sciences at the University of Campinas for analysis.

The sequence quality control and polymorphism analysis were performed using chromatograms. For multiple alignments, Geneious software (v. 6.0.6, Biomatters, New Zealand) was employed. Nucleotide sequences obtained from the National Center for Biotechnology Information database were aligned with sequences isolated using the Clustal W plug-in. The generated sequences were submitted to GenBank.

#### 4.4.2. Phylogenetic Analysis

Phylogenetic analyses were conducted using PAUP* software (v. 4.0b10, Sinauer Associates, UK). An unweighted parsimony analysis was executed through a heuristic search with 1000 random addition sequences and a tree-bisection-reconnection algorithm for branch swapping [42]. The consistency index (CI) and retention index (RI) were calculated to quantify homoplasy in the alignments. Clade stability was evaluated by Maximum Parsimony Bootstrap Proportions in PAUP*, using 1000 heuristic replications. For each phylogeny, species external to the analyzed group were used as the outgroup, and the trees were visualized with FigTree v. 1.4 [43].

## 5. Conclusions

Raw Brazil nut samples were evaluated for AFL occurrence and the impact of severe drought, revealing AFL concentrations exceeding those previously reported. Toxigenic strains were isolated from positive samples and identified through molecular sequencing. Phylogenetic analysis revealed four isolates belonging to *P. citrinum*. Morphological and sequencing analyses identified species from two sections of the genus *Aspergillus*: *Circumdati* and *Flavi*. These strains are also associated with other crops impacted by climate change, indicating that enhanced environmental protection measures throughout the Brazil nut supply chain must be implemented at various storage stages to mitigate AFL production and contamination. Considering that aflatoxigenic strains are susceptible to producing mycotoxins depending on variables such as environmental RH% and food MC, it is crucial to implement preventive measures to avoid contamination. Good management practices include controlling aeration during storage and protecting seeds from rain or excessive heat. They are cost-effective methods that can be established early in the production process without requiring large technological investments. Preventive measures help ensure the safety of raw materials for delivery to processing facilities and enhance sanitary standards. Future studies could investigate other variables that influence the metabolism of the studied strains associated with climate change in the Amazon, such as the efficacy of exhaust systems in storage facilities or the use of remote monitoring with dataloggers or real-time recording systems throughout the initial stages of the production chain.

## Figures and Tables

**Table 1:** Total aflatoxin concentration of the raw Brazil nuts collected during the 2023 extreme drought.

Harvest	Aflatoxins µg/kg Mean ± SD (Range)					Samples >Legal Limit *	Positive AFL Samples
	B1	B2	G1	G2	Total		
2023	3.22 ± 3.3 (0.39–9.16)	0.96 ± 1.7 (Nd–4.45)	3.28 ± 1.82 (1.55–6.59)	1.07 ± 0.16 (0.94–1.09)	8.54 ± 5.4 (3.19–16.53)	10%	100%

SD: Standard deviation; Nd: not detectable at limit of detection and limit of quantification for AFB1, AFB2, AFG1, and AFG2; 0.136, 0.136, 0.250, 0.250 μg/kg and 0.410, 0.410, 0.750, 0.750 μg/kg, respectively. * Samples above the legal limit [20].

**Table 2:** Water activity and moisture content % of raw Brazil nuts collected during the extreme drought of 2023.

	Water Activity			Moisture Content (%)		
	Mean ± SD	Range	>0.7 *	Mean ± SD	Range	>15 **
2023	0.77 ± 0.65	0.60–0.93	90%	4.77 ± 1.5	2.6–7.7	0%

SD: Standard deviation. * Water activity above the recommended limit t of 0.7. ** Moisture content by the Brazilian legal limit of 15%.

**Table 3:** Primer sets for ITS and β-tubulin *BenA* loci.

Locus	Primer	Sequence (5′-3′)	Reference
*ITS*	*ITS 1*	TCCGTAGGTGAACCTGCGG	White et al. [40]
	*ITS 4*	TCCTCCGCTTATTGATATGC	
*BenA*	*BT2A*	GGTAACCAAATCGGTGCTGCTTTC	Glass and Donaldson [41]
	*BT2B*	ACCCTCAGTGTAGTGACCCTTGGC	

## Data Availability

The original contributions presented in this study are included in the article. Further inquiries can be directed to the corresponding author.
